# Facile One-Step Microwave-Assisted Route towards Ni Nanospheres/Reduced Graphene Oxide Hybrids for Non-Enzymatic Glucose Sensing

**DOI:** 10.3390/s120404860

**Published:** 2012-04-13

**Authors:** Zhigang Wang, Yong Hu, Wenlong Yang, Mojiao Zhou, Xiao Hu

**Affiliations:** 1 Institute of Physical Chemistry, Zhejiang Normal University, Jinhua 321004, China; E-Mails: zhigangwang@zjnu.net (Z.W.); yangwenlong@zjnu.net (W.Y.); zhoumojiao@zjnu.net (M.Z.); 2 School of Materials Science & Engineering, Nanyang Technological University, 639798, Singapore

**Keywords:** carbon materials, nanocomposites, microwave-assisted method, sensors

## Abstract

In this work, a facile one-step microwave-assisted method for deposition of monodisperse Ni nanospheres on reduced graphene oxide (rGO) sheets to form Ni-rGO nanohybrids is discussed. In the presence of hydrazine monohydrate, Ni nanospheres are grown onto rGO sheets using nickel precursor and GO as starting materials in ethylene glycol (EG) solution under a low level of microwave irradiation (300 W) for 20 min, during which GO is also reduced to rGO. The as-prepared nanohybrids exhibit well-dispersed Ni nanosphere (about 80 nm in diameter) loadings and effective reduction of graphene oxide. The resulting Ni-rGO nanohybrids-modified glassy carbon electrode (GCE) shows significantly improved electrochemical performance in nonenzymatic amperometric glucose detection. In addition, interference from the oxidation of common interfering species under physiological conditions, such as ascorbic acid (AA) and uric acid (UA), is effectively avoided.

## 1. Introduction

The development of fast and reliable methods for glucose detection is of considerable importance due to its extremely important applications in clinical diagnosis, food analysis, and bioreactor monitoring [1,2]. Up to now, the glucose oxidase (GOD)-based sensors with high selectivity have been widely studied [3–5], but GOD-based biosensors suffer from a stability problem as the enzyme can be easily affected by temperature, pH value, humidity and toxic chemicals [6]. To overcome these obstacles, there is great interest in the fabrication of nonenzymatic glucose sensors based on the direct oxidation of glucose for extended usage. Compared with GOD-based biosensors, the nonenzymatic glucose sensors possess higher sensibility, lower detection limits, less susceptibility to environmental factors, better stability and shorter response times [7–9].

Recently, Ni-based materials have been extensively investigated for electrocatalytic oxidation of glucose, since they could allow production of glucose sensors in large numbers at low cost [10–14]. The oxidation processes are catalyzed by the Ni-based materials through the formation of a high-valent, oxyhydroxide species [NiOOH] in alkaline medium [11]. Graphene, a single layer of carbon atoms tightly packed into a two-dimensional [2D] honeycomb sp^2^ carbon lattice, has a unique ability to promote fast electron transfer kinetics for a wide range of electroactive species [15,16]. Deposition of metal nanoparticles on graphene sheets gives rise to nanocomposites with larger active surface areas and enhanced electron transport, making the nanocomposites ideal materials for the fabrication of electrochemical sensing devices [17]. It has been reported that graphene may play a distinct role in improving the conductivity of Ni-based materials for glucose sensors, in which the electron transfer is quick, effectively enhancing the detecting sensitivity and shortening the response times [18–20]. Very recently, chemical vapor deposition has increased in popularity in the synthesis of graphene/Ni nanohybrids, although the processes are complicated and hence difficult to scale [21,22].

**Scheme 1.**
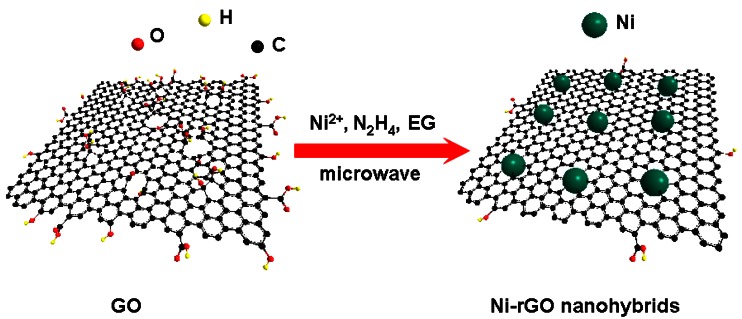
Schematic illustration of the formation of Ni-rGO nanohybrids via a facile one-step solvothermal route at low temperature.

Microwave irradiation is an attractive and facile method for the rapid synthesis of nanocrystals with small particle size, narrow particle size distribution, and high purity. Compared with conventional heating, it has a more homogeneous heating process. Moreover, it can promote nucleation and reduce the synthesis times considerably, generating smaller and more uniform particles [23–25]. Herein, we demonstrated a facile one-step microwave-assisted method to directly deposit Ni nanospheres on reduced graphene oxide (rGO) sheets (Scheme 1). When a GO sheet solution is mixed with a nickel salt solution, Ni^2+^ is selectively bonded with carboxyl through mutual electrostatic attraction. Under continuous stirring conditions, the interlayer spacing gradually increases and Ni^2+^ could interlaminate more easily into the enlarged layer [26]. In the presence of hydrazine monohydrate, Ni nanospheres were grown onto rGO sheets in ethylene glycol (EG) solution under a low level of microwave irradiation (300 W) for 20 min, during which GOs were also reduced to rGO. These nanocomposites exhibit well-dispersed Ni nanosphere (about 80 nm in diameter) loadings and effective reduction of graphene oxide. By forming the rGO-supported Ni nanospheres (Ni-rGO nanohybrids), one might be able to take advantage of the best features of both components. As expected, the as-prepared Ni-rGO nanohybrid-modified glassy carbon electrode (GCE) shows highly sensitivity and fast amperometric sensing of glucose. In addition, interference from the oxidation of common interfering species present in body fluids, such as ascorbic acid (AA) and uric acid (UA), is effectively avoided.

## 2. Experimental Details

All reagents were of analytical grade, purchased from the Shanghai Chemical Reagent Manufacturing Co., and used as received without further purification.

### 2.1. Preparation of GO

In a typical procedure, GO was first prepared from pure graphite using a modified Hummer's method [27,28]. Briefly, 2.0 g of natural graphite powder was added to 300 mL of H_2_SO_4_ under stirring at 0 °C, and then 3.0 g of NaNO_3_ and 20 g of KMnO_4_ were added gradually. Successively, the mixture was transferred to a water bath at 30 °C and stirred for 20 min to form a thick paste. Then, 250 mL of distilled water was slowly added and the temperature was increased to 98 °C. After 30 min aging, another 500 mL of water was added and this was followed by a dropwise addition of 40 mL of H_2_O_2_ (30%). When the color of the solution changed from dark brown to brilliant yellow, the mixture was filtered and washed with diluted HCl aqueous (1/10 v/v) three times to remove metal ions, and then washed with distilled water repeatedly until the pH became 7. Finally, the as-prepared GO was obtained after drying in a vacuum oven at room temperature.

### 2.2. One-Step Microwave-Assisted Growth of Ni Nanospheres on rGO Sheets

Next, 0.058 g of Ni(NO_3_)_2_·6H_2_O and 10 mg of the as-prepared GO were dissolved in 40 mL of EG with the assistance of ultrasonication for 1 h. Subsequently, the mixture was transferred to an oil bath under vigorous stirring, and then 2 mL of hydrazine monohydrate (N_2_H_4_·H_2_O, 85%) was slowly added and the solution was placed in a microwave refluxing system irradiated at 300 W for 20 min. Finally, the as-prepared products were collected and thoroughly rinsed several times with distilled water and ethanol and then dried at 60 °C for 12 h.

### 2.3. Characterization

Powder X-ray diffraction (XRD) measurements of the samples were performed with a Philips PW3040/60 X-ray diffractometer using Cu_Kα_ radiation at a scanning rate of 0.06°s^−1^. Fourier transform infrared (FT-IR) spectra were recorded on a Nicolet NEXUS670 FT-IR spectrometer using KBr pellets. Scanning electron microscopy (SEM) was performed with a Hitachi S-4800 scanning electron microanalyzer with an accelerating voltage of 15 kV. Transmission electron microscopy (TEM) and high resolution TEM were conducted at 200 kV with a JEM-2100F field emission TEM, after dispersing the Ni-rGO nanohybrids in ethanol and depositing several drops of the suspension on the carbon coated copper grids and dried under ambient conditions.

### 2.4. Electrochemical Measurements

All electrochemical measurements were conducted using a CHI840C electrochemical workstation with conventional three-electrode setup at room temperature. A Ni-rGO hybrids modified GCEs was employed as working electrode, a saturated Hg/Hg_2_Cl_2_ electrode (SCE) as reference electrode and platinum wire as counter electrode. The supporting electrolyte is 0.1 M NaOH containing 0.1 M KCl, and deionized water was used throughout the experiments. For calibration experiment, amperometric measurements were carried out at 0.5 V, while 100 μL of 0.2 or 2 mM glucose was added into 20 mL of electrolyte under a magnetically stirred condition to obtain a stepwise increase to reach a final concentration 1 μM or 10 μM.

## 3. Results and Discussion

**Figure 1.**
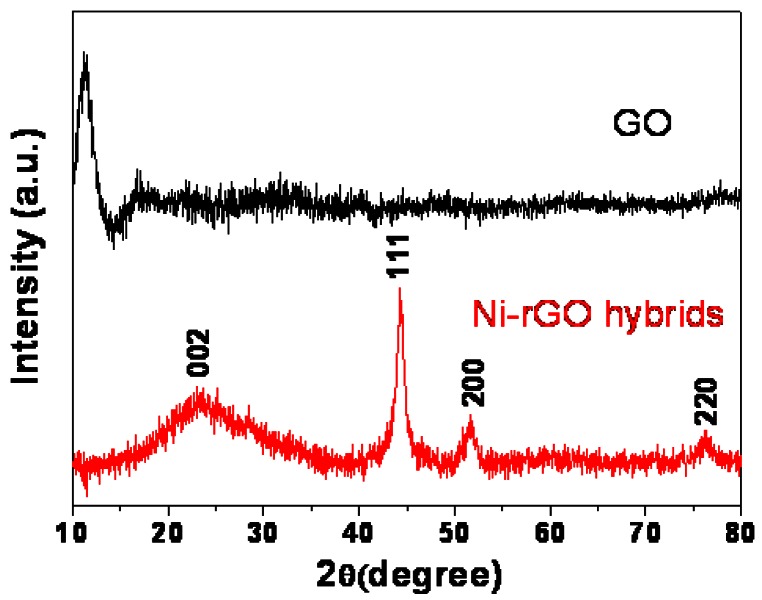
XRD patterns of the as-prepared GO and Ni-rGO nanohybrids.

The XRD patterns of the as-prepared GO and Ni-rGO nanohybrids are shown in Figure 1. For GO, the characteristic peak at around 11.3° (the d-spacing is about 0.79 nm) is consistent with the interlayer spacing of GO sheets reported previously, which may be ascribed to the existence of oxygen-rich groups on both sides of the sheets and water molecules trapped between the sheets [29].

In the XRD pattern of the Ni-rGO nanohybrids, the clear diffraction bands centered at 2θ of 44.4°, 51.8°, and 76.3° are corresponding to the (111), (200), and (220) crystal planes respectively of the face-centered cubic (fcc) Ni (JCPDS 04-0850, a = 0.3523 nm). A broad hump at 23.2° can be attributed to the (002) diffraction of rGO, indicating the successfully reduction of GO by hydrazine hydrate [30]. In addition, the average size of Ni nanoparticles is 9.4 nm, which calculated using Debye-Scherrer equation based on the full width at half-maximum of the diffraction peak.

**Figure 2.**
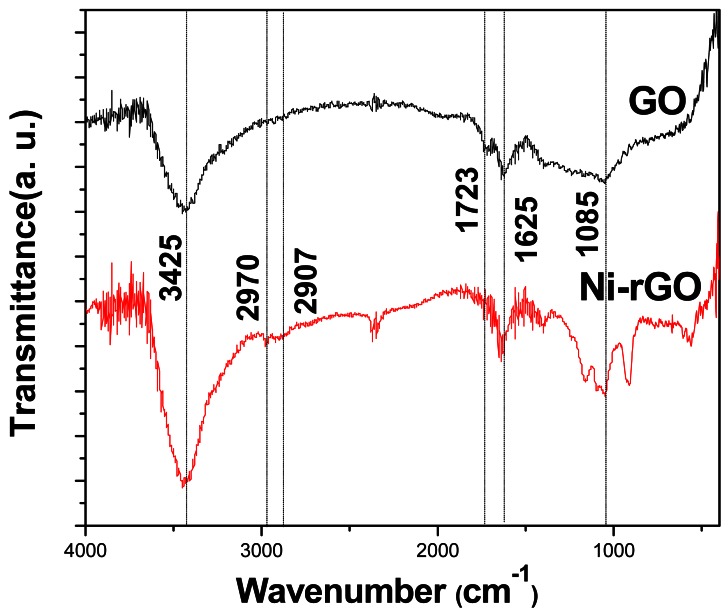
FT-IR spectra of the as-prepared GO and Ni-rGO nanohybrids.

Figure 2 shows the FT-IR spectra of the as-prepared of GO and Ni-rGO nanohybrids. The broad absorptions at about 3,425 and 1,625 cm^−1^ are assigned to the hydroxyl groups of absorbed H_2_O molecules, and the peaks around 2,970 and 2,900 cm^−1^ can be assigned to the asymmetric and symmetric vibrations of C-H, respectively. The absorption band at 1,087 cm^−1^ can be assigned to the stretching vibration of C-O. The C=O vibration band at 1,723 cm^−1^ disappears after hydrazine hydrate reduction of exfoliated GO, indicating that the reduction of GO to rGO is complete [31].

**Figure 3.**
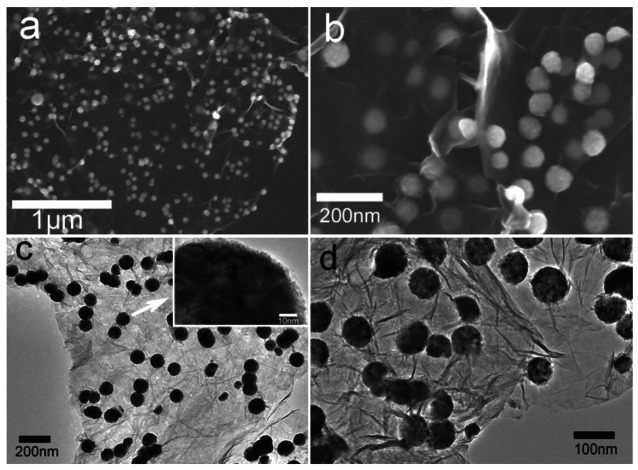
(**a**) Low-magnification and (**b**) high-magnification SEM image of the as-prepared Ni-rGO nanohybrids; (**c**) Low-magnification and (**d**) high-magnification TEM image of the as-prepared Ni-rGO nanohybrids.

The SEM images reveal the morphology of the Ni-rGO nanohybrids prepared by our one-step microwave-assisted method. The low magnification SEM image (Figure 3(a)), reveals that the GO sheets are well exfoliated and nearly monodispersed Ni nanospheres are anchored uniformly on the surface of the rGO sheets. Observation under higher magnification (Figure 3(b)) shows the well-dispersed Ni spheres with a diameter of about 80 nm on the wrinkly rGO sheets. The transmission electron microcopy (TEM) images (Figure 3(c,d)) also show that these monodiperse spheres are about 80 nm in size, while the HRTEM image (inset in Figure 3(c)) indicates that there are smaller nanocrystals attached uniformly to form nanospheres. In our study, hydrazine does not only acts as a reducing agent to form Ni nanospheres in the subsequent solvothermal process, but also plays an important role in reducing GO to rGO sheets. GO, bearing hydroxyl and epoxide groups on the basal planes, along with carbonyl and carboxyl groups along the sheet edges, readily coordinates with Ni^2+^ from solution. The strong reducing ability of hydrazine hydrate ensures synchronous reduction of GO and nickel ions which leads to the formation of nanohybrids [32]. Additionally, we believe the EG solvent selected for this experiment also acts as a surfactant [10], which absorbs on the GO surface, while at the same time providing hydrophilic hydroxyl groups for the hydrolysis of the nickel precursor. Hence, GO can be used as a template for deposition of Ni nanospheres on its surface.

**Figure 4.**
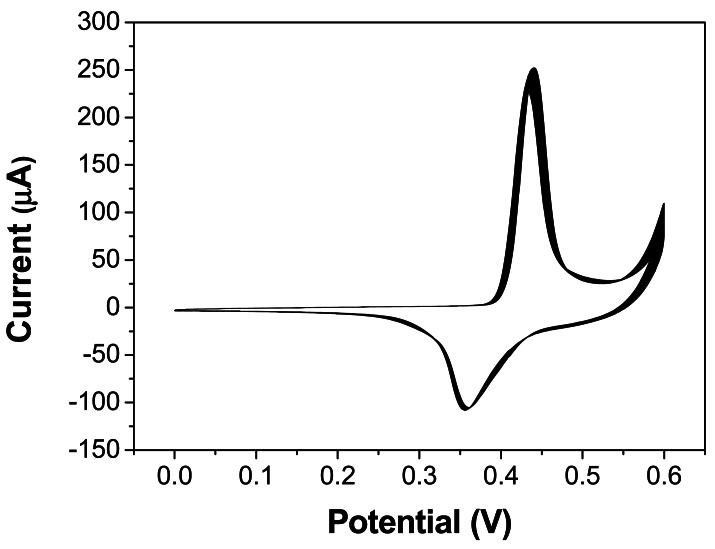
Forty consecutive cyclic voltammograms curves of the N-rGO nanohybrid- modified electrode in 0.1 M NaOH solution at a scan rate of 0.1 Vs^−1^.

To investigate the electrocatalytic activity of the as-prepared Ni-rGO hybrid-modified GCE toward the oxidation of glucose, the corresponding cyclic voltammograms (CVs) were obtained in the absence and presence of glucose in 0.1 M of NaOH solution at a potential sweep rate of 0.1 Vs^−1^. Figure 4 shows the CVs of the as-prepared GO and Ni-rGO hybrid-modified GCE in the 0.1 M NaOH solution in the absence glucose and in the presence of 1 mM glucose. There is no obvious reaction peak current observed for the GO modified GCEs either in the absence or presence of glucose. However, a pair of well-defined redox peaks with a cathodic peak at 361 mV and the corresponding anodic peak at 441 mV is observed at the Ni-rGO hybrid-modified GCE in the absence of glucose, which are assigned to the Ni(III)/Ni(II) redox couple [12,33].

The Ni (II) species on the electrode are due to the oxidation of Ni(0). Upon addition of 1 mM glucose, there is a great enhancement of the anodic peak current and considerable decrease of cathodic peak current, which indicates that the Ni-rGO hybrids could catalyze the oxidation of glucose. The oxidation of glucose to glucolactone is catalyzed by the Ni(III)/Ni(II) redox couple, where the Ni(III) species on the electrode surface rapidly oxidizes glucose at the anode, converting Ni(II) into Ni(III) species. Thus, the change in concentrations of Ni(II) and Ni(III) ratio causes the increase of the anodic peak current and the decrease of the cathodic peak current [11]. In addition, the stability for the Ni-rGO hybrid-modified GCE after activation is also examined by recording 40 consecutive CV curves between 0 and 600 mV in 0.1 M NaOH at a scan rate of 0.1 Vs^−1^ (Figure 4). No obvious peak current change is observed, which demonstrates the Ni-rGO hybrid- modified GCE is very stable.

**Figure 5.**
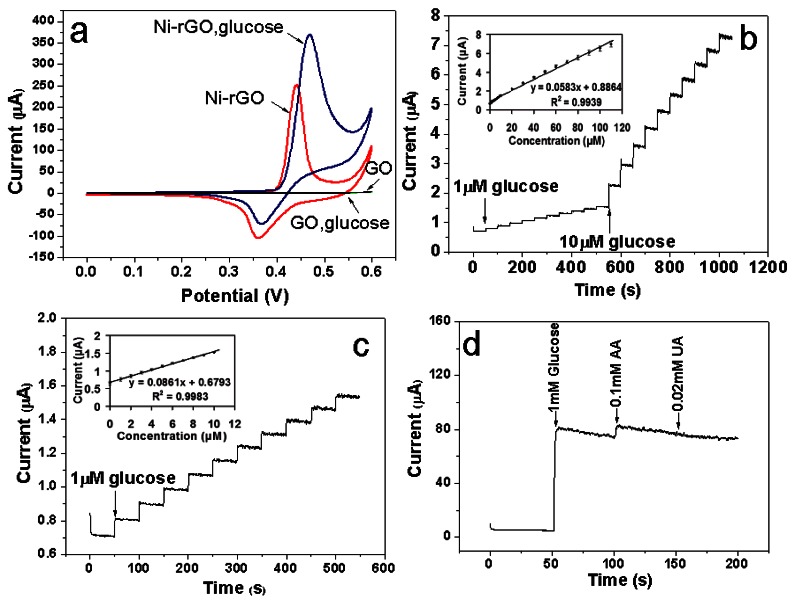
(**a**) CVs of the as-prepared GO and Ni-rGO nanohybrids modified GCE recorded in 0.1M NaOH solution and in 0.1M NaOH solution and in the presence of 1 mM glucose (Scan rate: 0.1 Vs^−1^), and the amperometric response of the Ni-rGO nanohybrid-modified GCE to successive addition of glucose from: (**b**) 1 to 110 μM, and (**c**) 1 to 10 μM into 0.1 M NaOH with stirring at an applied potential of 0.5 V. The linear relationship between the catalytic current and glucose concentration were inset, respectively. (**d**) Successive injection of 1 mM glucose and interfering species 0.1 mM AA and 0.02 mM UA at 100 s intervals into 0.1 M NaOH at an applied potential of 0.5 V.

Figure 5(b) shows the well-defined steady-state amperometric response of the as-prepared Ni-rGO hybrid-modified GCE nonenzymatic glucose sensing with the successive addition of glucose to 0.1 M NaOH at an applied potential of 0.5 V.

The response time is less than 5 s, revealing a rapid and sensitive response to glucose, which is ascribed to the excellent electrocatalytic properties and rapid charge transfer of the Ni-rGO hybrid electrodes. The calibration plot (inset in Figure 5(b)) has a wide linear range between 1 and 110 μM with a gradient of 57.5 nA·μM^−1^ and a correlation coefficient of 0.9939 (the sensitivity is about 813 μA·mM^−1^·cm^−2^). More detailed examination of Figure 5(c) reveals the linear relationship with a better correlation coefficient of 0.9983 (inset in Figure 5(c)) over the lower concentration range of 1–10 μM. In this case, there is a steeper gradient of 66.2 nA·μM^−1^ corresponding to a higher sensitivity of about 937 μA·mM^−1^·cm^−2^. The oxidation of glucose on the Ni surface can be regarded as a direct electrooxidation process. The coverage of Ni(III) on the Ni surface increases with the increase of potential. There are enough glucose molecules to adsorb on the Ni(III) sites initially, and which is an adsorption-controlled process that is not sensitive to the concentration. When the potential is high enough, the glucose diffusing to the electrode surface cannot inhabit the active Ni(III) sites completely, and which is a diffusion controlled process that is sensitive to the concentration. There is a potential at which the glucose molecules diffusing to the electrode are equal to the amount of Ni(III) sites. It is obviously that a higher potential is needed to form more Ni(III) sites for a higher concentration of glucose. The anodic peak current then shifts positively as the glucose concentration increases [34]. Thus, the Ni-rGO hybrid-modified GCE possesses better sensitivity for lower concentrations of glucose.

Furthermore, it is well known that AA and UA present in the biological samples could be easily oxidized at positive potential and often interfere with the detection of glucose by other methods [35]. To evaluate the selectivity of this sensor, the amperometric response of the Ni-rGO hybrid-modified electrode to UA (0.02 mM), AA (0.1 mM), and glucose (1 mM) on the physiological level at an applied potential of 0.5 V in 0.1 M NaOH solution is shown in Figure 5d. Compared with glucose, the current responses of these interfering species are very weak and can be neglected. Therefore, the as-prepared Ni-rGO hybrid-modified GCE also exhibits excellent selectivity for glucose detection.

## 4. Conclusions

In summary, we have presented a new and facile method to prepare hybrid nanostructures of Ni nanospheres deposited onto rGO sheets (Ni-rGO), by combining Ni precipitation and GO reduction in one single step via a rapid microwave-assisted process. The Ni nanospheres are uniform in size and well-dispersed onto the rGO surfaces. The GCE electrodes modified using these Ni-rGO hybrids exhibited high sensitivity, fast response and excellent stability for nonenzymatic determination of glucose. The simple preparation procedures, low cost and enhanced electrocatalytic activity can potentially pave the way for inexpensive, effective and highly sensitive glucose sensors.
